# Mitochondrial haplogroups modify the effect of black carbon on age-related cognitive impairment

**DOI:** 10.1186/1476-069X-13-42

**Published:** 2014-05-30

**Authors:** Elena Colicino, Melinda C Power, David G Cox, Marc G Weisskopf, Lifang Hou, Stacy E Alexeeff, Marco Sanchez-Guerra, Pantel Vokonas, Avron Spiro III, Joel Schwartz, Andrea A Baccarelli

**Affiliations:** 1 Department of Environmental Health, Harvard School of Public Health, 665 Huntington Ave, Boston, MA 02115, USA; 2 Department of Epidemiology, Harvard School of Public Health, 665 Huntington Ave, Boston, MA 02115, USA; 3 INSERM U1052, Centre de Recherche en Cancérologie de Lyon, Lyon F-69000, France; 4 Centre Léon Bérard, Pole de Recherche Translationnelle, Lyon F-69008, France; 5 Department of Preventive Medicine, Northwestern University Feinberg School of Medicine, 420 East Superior St, Chicago, IL 60611, USA; 6 VA Boston Healthcare System, Boston University Schools of Public Health and Medicine, 88E Newton St, Boston, MA 02118, USA

**Keywords:** mtDNA haplogroups, Air pollution, Black carbon, Cognitive decline, Mini-mental state examination

## Abstract

**Background:**

Traffic-related air pollution has been linked with impaired cognition in older adults, possibly due to effects of oxidative stress on the brain. Mitochondria are the main source of cellular oxidation. Haplogroups in mitochondrial DNA (mtDNA) mark individual differences in oxidative potential and are possible determinants of neurodegeneration. The aim of this study was to investigate whether mtDNA haplogroups determined differential susceptibility to cognitive effects of long-term exposure to black carbon (BC), a marker of traffic-related air pollution.

**Methods:**

We investigated 582 older men (72 ± 7 years) in the VA Normative Aging Study cohort with ≤4 visits per participant (1.8 in average) between 1995–2007. Low (≤25) Mini Mental State Examination (MMSE) was used to assess impaired cognition in multiple domains. We fitted repeated-measure logistic regression using validated-LUR BC estimated in the year before their first visit at the participant’s address.

**Results:**

Mitochondrial haplotyping identified nine haplogroups phylogenetically categorized in four clusters. BC showed larger effect on MMSE in Cluster 4 carriers, including I, W and X haplogroups, [OR = 2.7; 95% CI (1.3-5.6)], moderate effect in Cluster 1, including J and T haplogroups [OR = 1.6; 95% CI: (0.9-2.9)], and no effect in Cluster 2 (H and V haplogroups) [OR = 1.1; 95% CI: (0.8-1.5)] or Cluster 3 (K and U haplogroups) [OR = 1.0; 95% CI: (0.6-1.6)]. BC effect varied only moderately across the I, X, and W haplogroups or across the J and T haplogroups.

**Conclusions:**

The association of BC with impaired cognition was worsened in carriers of phylogenetically-related mtDNA haplogroups in Cluster 4. No BC effects were detected in Cluster 2 and 3 carriers. MtDNA haplotypes may modify individual susceptibility to the particle cognitive effects.

## Background

Recent epidemiologic investigations have linked age-related loss of cognition to long-term exposure to particulate air pollution [1-3]. A form of particulate matter is Black Carbon (BC), which is often used as a marker of air pollution from traffic sources. BC exposure has been specifically associated with lower cognitive performance [3]. The pathways linking traffic-related air pollution to cognitive impairment have not yet been thoroughly clarified [4].

Mitochondria are membrane-enclosed organelles that perform metabolic reactions to create energy in the form of Adenosine-5′-triphosphate (ATP) through multi-enzyme complexes located in both the inner mitochondrial membrane and the mitochondrial matrix [5]. Mitochondrial dysfunction has been linked to human age-related and neurogenerative disorders, due at least in part to augmented mitochondrial reactive oxygen species production [6]. Because air pollution effects on cognition have been suggested to be mediated through generation of oxidative species [7], markers of mitochondrial dysfunction may help clarify the mechanisms activated by the exposure and identify individuals at higher risk of exposure-related cognitive impairment. Mitochondria have their own DNA (mtDNA) enclosed in a single circular chromosome that is inherited through the maternal lineage. This mtDNA codes for 13 essential polypeptide components of the mitochondrial respiratory chain, two ribosomal Ribonucleic acids (RNA)s, and 22 transfer RNAs. mtDNA has a higher mutation rate than nuclear DNA [8]. Consequently, a considerable number of mtDNA point mutations (single-nucleotide polymorphisms) have accumulated sequentially along maternal lineages [9]. A number of stable polymorphic sites have been identified in mtDNA coding regions that define related groups of mtDNA, called haplogroups [9]. The haplogroups may be further grouped into clusters according to their phylogenetic background [9]. Because of the relevance of mtDNA for energy production and oxidative stress generation, mtDNA haplogroups and clusters have been intensively investigated in relation to aging and neurodegerative diseases characterized or accompanied by cognitive impairment, including Alzheimer’s disease [10], and frontal lobe disease [11].

In the present study, we haplotyped participants in a large prospective cohort of aging and categorized them according to clusters of mtDNA haplogroups. We hypothesized that mtDNA haplogroups modified the effects of BC on cognitive function.

## Methods

### Study sample

The US Department of Veterans Affairs (VA) Normative Aging Study (NAS) is an ongoing longitudinal cohort established in 1963, which included men who were 21–80 years of age and free of known chronic medical conditions at entry [12,13]. Participants were subsequently invited to medical examinations every three to five years. Starting in 1993, all participants completed a battery of cognitive tests. We excluded participants who had experienced a stroke (3% of individuals), leaving a total of 582 individuals with complete BC, mtDNA haplogroup, and covariate data. The study was approved by the Institutional Review Boards (IRB)s of the participating institutions. Participants have provided written informed consent at each visit. Counts of eligible and non-eligible participants and reason for ineligibility are reported in Additional file 1: Table S1.

### Cognitive testing

We considered the Mini-Mental State Examination (MMSE), which was administrated with consistency over the period 1995–2007 among the cohort participants. MMSE is a test of global cognition that assesses multiple cognitive areas, i.e., orientation, immediate and short-term recall, attention and calculation, word finding, figure construction, reading and writing skills, and ability to follow a 3-step command. The range of scores is 0 to 30. The MMSE is extensively validated and used in clinical practice and research, as well as in epidemiology, as a dementia screening [14]. In this study, the maximum MMSE score was 29, due to the exclusion of the MMSE question on the county of residence, which has limited political and administrative meaning in Massachusetts and thus not of diagnostic utility [14,15]. We included cognitive data from study visits performed since 1995 (i.e., the first year after a full year of BC exposure data could be estimated) through 2007. We defined as the baseline visit for each study participant the first cognitive assessment completed on or after July 1st, 1995. Up to 4 cognitive testing were completed by the study participants and were all used in the statistical analysis. On average each participant completed 1.8 tests.

### Exposure assessment

A validated spatio-temporal land-use regression model in the greater Boston, Massachusetts area was used to obtain daily estimates since 1994 of Black Carbon (BC) exposure at the residence of each participant [3,16]. The model develops daily BC predictions from daily BC estimates averaged from 83 monitoring sites. Included in the model were predictors based on meteorological conditions (e.g., wind speed), land use (e.g., traffic density), daily BC concentrations at a central monitor, and other descriptors (e.g., day of the week). The goodness of fit of the model, based on the training set, was high (R^2^ = 0.83) and the average correlation between predicted values and observed BC levels in four out-of-sample validation samples was moderate (average R^2^ = 0.59). For the present study, addresses with predicted daily BC concentrations outside the range of the exposure measurements from the training set were excluded. We considered as a metric of long-term exposure the average of the 365 daily estimates at the participant’s residential address before the date of the baseline cognitive assessment of each participant [3].

### Mitochondrial (mtDNA) haplogroups

**Figure 1:**
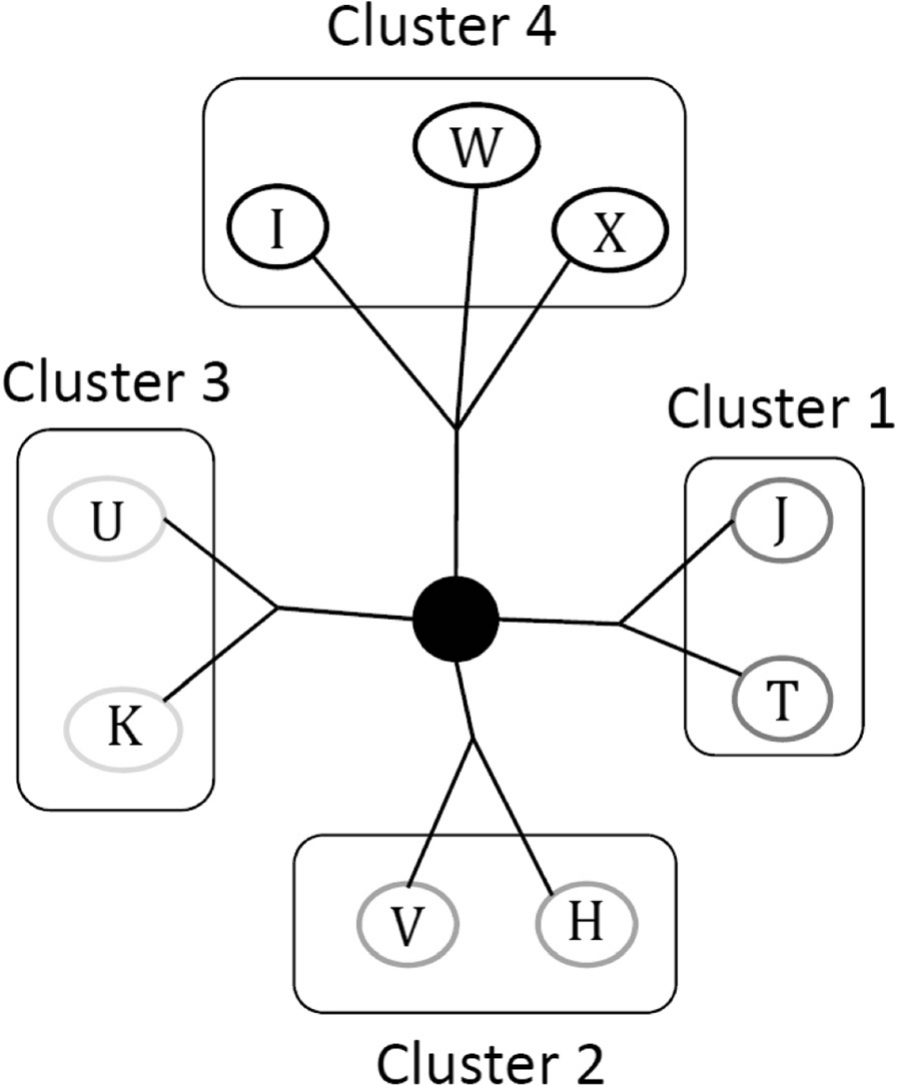
Position on a simplified phylogenetic evolutionary tree of the mtDNA haplogroups and clusters in NAS.

Genotyping was conducted on blood DNA using Taqman assays (Applied Biosystems, Foster City, CA) for all polymorphisms, with the exception of 8994 which was genotyped using Sequenom due to Taqman design failure. Primer and probe sequences, as well as cycling conditions are available upon request. Internal blinded quality control samples were >99% concordant, and all samples yielded genotypes. Hardy-Weinberg equilibrium tests are not valid for mitochondrial SNPs, and therefore were not assessed. No heteroplasmy (detected as heterozygous samples) was observed. The mtDNA haplogroups found in the cohort were H, I, J, K, T, U, V, W, and X, which are most common in populations of European ancestry (Figure 1). To limit multiple comparison testing, haplogroups were grouped into four clusters according to the phylogenetic evolutionary tree [9,11]. Because haplogroups are inherited through maternal lineages, matrilineal ethnicity was collected through questionnaire and categorized based on the self-reported maternal grandmother’s ancestry. Four-hundred-fifty-two individuals did not provide blood DNA for the haplogroup analysis and were therefore excluded from the study. The participants without haplogroup information were similar to the eligible participants for BC levels and MMSE scores (Additional file 1: Table S2).

### Statistical analysis

A consistent proportion of participants (14% of our observations) achieved the maximum score in the MMSE, thus revealing a ceiling effect for MMSE scores. Only 8% of our observations exhibited scores ≤24, i.e. the typical screening cut-off score used in research on dementia. Therefore we considered scores ≤25 as low performance of MMSE (19% of our observations) [3], created a dichotomous variable for low MMSE performance based on this cutoff, and used this in all models as the dependent variable.

We first evaluated the main effect of mtDNA clusters, as well as of BC, on the odds of having a low MMSE score. We then evaluated whether the BC-cognition association was modified by clusters of mtDNA haplogroups. For clusters of mtDNA haplogroups that showed main effects or interactions, we planned on also evaluating individual haplogroups within each cluster. In all models, we used logistic regression with generalized estimating equations and empirical variance estimates to account for the repeated measures for each individual. All models were adjusted for potential confounders or predictors of cognitive function, including age at cognitive assessment (as a continuous variable) and several variables at baseline, i.e. education (<12, 12|–|16, >16 years), matrilineal ethnicity (English speaking European ancestry, Mediterranean Europe, other European countries, other countries), first language (English/not English), computer experience (yes/no), smoking (current, former, never), body mass index (BMI) (<25, ≥ 25), physical activity (<12, 12|-30, ≥ 30 metabolic equivalent hours (MET-hr) per week), alcohol intake (<2 drinks/day, ≥2 drinks/day), diabetes (yes/no), hypertension (yes/no), dark fish consumption (<once a week, ≥once a week), percentage of the participant’s census tract that is nonwhite, percentage of residential census tract adults with a college degree, indicator for first cognitive assessment (yes/no) to differentiate from the following assessments, and an indicator for whether the participant was a part-time resident of the greater Boston area (yes/no). Education, computer experience and dark fish consumption also served as proxies of SES and diet quality for each participant.

A sensitivity analysis was conducted adjusting for only age and both age and education at the baseline.

A change in cognitive function was also examined to complete the analysis. The dependent variable consisted in the difference between the MMSE score at the second, third and fourth visit and the first cognitive assessment. We used linear regression models with generalized estimating equations and empirical variance estimates. The potential confounders or predictors of the change of cognitive function included in the model were the same baseline variables described above, as well as age at baseline and the age difference between age at the visit and age at baseline.

Because of the log-linear relationship between BC and MMSE, as indicated by restricted cubic splines [3], a log-transformation of BC was used in the all analyses. We used SAS (version 9.2; SAS Institute Inc., Cary, NC) for all analyses.

## Results

### Study participants characteristics

The mean age of the study participants at baseline was 72 years (Standard Deviation (SD) 7; range, 53–97 years). Most men reported English as their first language (86%) and alcohol consumption <2 drinks per day (75%), were current or former smokers (70%) and did not have diabetes mellitus (86%). A minority were of normal adiposity (BMI < 25; 23%) or did not have hypertension (33%) (Table 1). On the natural scale, 1-year average BC exposure estimates ranged between 0.03–1.77 μg/m^3^ (mean ± SD, 0.6 ± 0.3 μg/m^3^) and exhibited a right skewed distribution (details in [3,16]). We log-transformed BC (ln(BC)) and reported associations for a doubling in BC concentration on the natural scale, or approximately a 0.7 unit change in ln(BC) (details in [3]). The distribution of MMSE performance by mitochondrial clusters and haplogroups was reported in Additional file 1: Table S3. MMSE was associated with most of the potential confounders and predictors (Additional file 1: Table S4).

**Table 1:** Baseline characteristics of the Normative Aging Study (NAS) cohort (N = 582)

**Characteristics**	**N**	**(%)**	**BC concentration (μg/m**^**3**^**) Mean ± StdDev**
**Age (Years)**			
50-59	38	(6.5)	0.54 ± 0.30
60-69	243	(41.8)	0.60 ± 0.28
70-79	242	(41.6)	0.59 ± 0.27
80-89	57	(9.8)	0.61 ± 0.35
90-99	2	(0.3)	0.47 ± 0.40
**Education (Years)**			
<12	175	(30.1)	0.63 ± 0.30
12|–|16	290	(49.8)	0.59 ± 0.28
>16	117	(20.1)	0.53 ± 0.24
**First Language**			
English	498	(85.6)	0.59 ± 0.28
Not English/Bilingual	84	(14.4)	0.60 ± 0.27
**Computer Experience**			
Yes	242	(41.6)	0.56 ± 0.26
No	340	(58.4)	0.61 ± 0.29
**Physical Activity (MET-hr/week)**			
<12	324	(55.7)	0.59 ± 0.29
12|–30	160	(27.5)	0.61 ± 0.29
≥30	98	(16.8)	0.57 ± 0.25
**Alcohol (drinks/day)**			
<2	439	(75.4)	0.59 ± 0.29
≥2	143	(24.6)	0.58 ± 0.25
**Diabetes**			
No	499	(85.7)	0.59 ± 0.29
Yes	83	(14.3)	0.60 ± 0.25
**Consumed Dark Fish (times/week)**			
<1	497	(85.4)	0.59 ± 0.28
≥1	85	(14.6)	0.57 ± 0.28
Continued on the next page			
Continued from the previous page			
**Nonwhite (% of census tract)**			
<5%	226	(38.8)	0.54 ± 0.30
5% |—10%	157	(27)	0.55 ± 0.25
≥10%	199	(34.2)	0.68 ± 0.26
**≥25 years of age with at least a college degree (% of census tract)**			
<30%	192	(33)	0.64 ± 0.30
30% |—50%	230	(39.5)	0.56 ± 0.28
≥50%	160	(27.5)	0.58 ± 0.25
**Smoking Status**			
Never	172	(29.6)	0.57 ± 0.25
Former	34	(5.8)	0.63 ± 0.21
Current	376	(64.6)	0.60 ± 0.30
**BMI (Kg/m**^**2**^**)**			
<25	136	(23.4)	0.61 ± 0.27
≥25	446	(76.6)	0.58 ± 0.28
**Hypertension**			
No	190	(32.6)	0.56 ± 0.26
Yes	392	(67.4)	0.61 ± 0.29
**Ethnicity**			
English Speaking European countries	263	(45.2)	0.61 ± 0.30
Mediterranean European countries	141	(24.2)	0.60 ± 0.27
Others European countries	113	(19.4)	0.55 ± 0.26
Others	65	(11.2)	0.56 ± 0.27

### Frequency of mitochondrial haplogroups and clusters

Cluster 1 included haplogroups J or T and was found in 19% of the participants. Cluster 2 included haplogroups H and V and was the most common among the participants (51%), and was therefore taken as the reference category. Cluster 3 included haplogroups K and U and was found in 20% of participants. Cluster 4 included the most ancestral haplogroups I, W, and X and was found in 10% of the participants (Table 2). MtDNA haplogroups reported significant association with matrilineal ethnicity, BMI, first language, SES variables (education, dark fish consumption, computer experience), diseases (hypertension, diabetes) and life style variables (smoking status, alcohol intake) (Additional file 1: Table S4).

**Table 2:** Proportion of the mitochondrial clusters and haplogroups in the Normative Aging Study (NAS) cohort participants (N = 582)

**Haplotypes**	**N**	**%**
**Cluster 1 (J or T)**	**108**	**19**
Haplogroup J	50	9
Haplogroup T	58	10
**Cluster 2 (H or V)**	**296**	**51**
Haplogroup H	51	9
Haplogroup V	245	42
**Cluster 3 (K or U)**	**116**	**20**
Haplogroup K	55	10
Haplogroup U	61	10
**Cluster 4 (I, W or X)**	**62**	**10**
Haplogroup I	32	5
Haplogroup W	8	1
Haplogroup X	22	4

### BC effects on cognition and modification by mtDNA haplogroups

**Figure 2:**
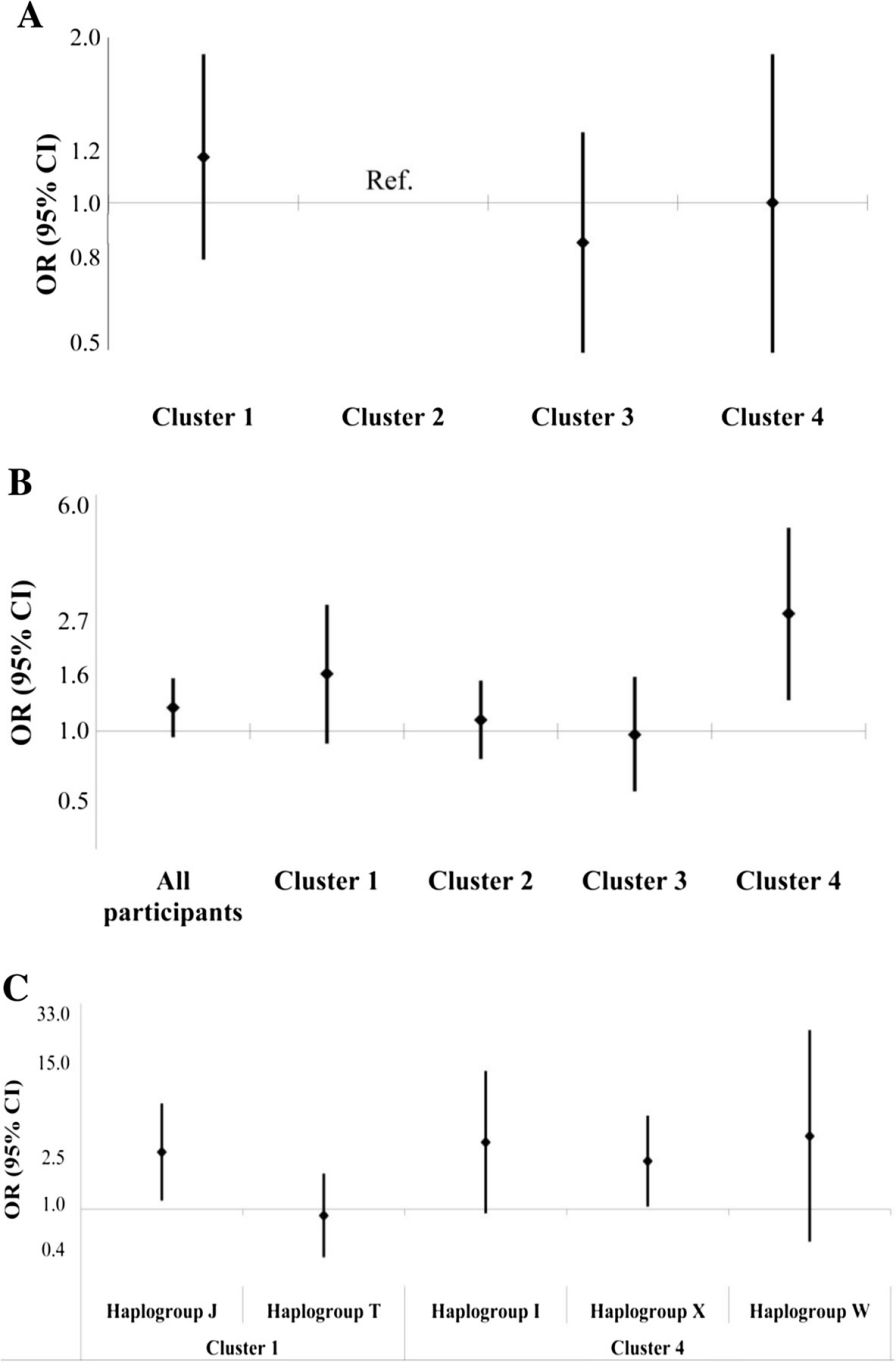
**Main effect and effect modification of clusters and mitochondrial haplogroups on cognitive function.** The figure shows the main effect of mtDNA haplogroups clusters on the low MMSE **(Panel A)**; effect for doubling in BC concentration on the natural scale on the low MMSE score overall and by mitochondrial clusters (the most common cluster, i.e. Cluster 2, was taken as reference) **(Panel B)**; and effect for doubling in BC concentration on the natural scale on the low MMSE score by individual haplogroups (the most common haplogroup, i.e. haplogroup V, was taken as reference) **(Panel C)**.

In the entire study population, each doubling in BC on the natural scale was associated with 1.22 times higher odds (95% Confidence Interval (CI): 0.95-1.56; Figure 2B; Additional file 1: Table S6) of low MMSE score adjusted for clinical and lifestyle factors. We examined the main effect of mtDNA haplogroup clusters on cognition by estimating the relative odds of low MMSE score relative to Cluster 2, taken as reference (Figure 2A; Additional file 1: Table S5). None of the clusters showed an association with low MMSE score.

BC effects on MMSE varied by mtDNA haplogroup clusters (Figure 2B; Additional file 1: Table S6). BC effect was larger among Cluster 4 carriers (Odds Ratio (OR) = 2.70 for a doubling in BC concentration; 95% CI: 1.30-5.59; OR for the interaction term = 2.46; 95% CI: 1.10-5.48). No effect of BC on MMSE was found in carriers of Cluster 2 (OR = 1.10 for a doubling in BC concentration; 95% CI: 0.79-1.53; Cluster 2 was the reference category for the interaction term) or Cluster 3 (OR = 0.97 for a doubling in BC concentration; 95% CI: 0.60-1.58; OR for the interaction = 0.89; 95% CI 0.50-1.57). A moderate borderline effect of BC on MMSE was found among Cluster 1 carriers (OR = 1.62 for a doubling in BC concentration; 95% CI: 0.90-2.91; OR for the interaction = 1.48; 95% CI: 0.77-2.86).

To further characterize Cluster 4 and 1, we evaluated the individual mtDNA haplogroups included in the clusters taking as reference haplogroup V, the most common haplogroup and part of Cluster 2 (Figure 2C; Additional file 1: Table S7). Estimates for the BC effects on low MMSE within each haplogroup had low precision, but they did not vary substantially across the three haplogroups in Cluster 4. In Cluster 1, we found larger BC effect in haplogroup J carriers (OR = 3.04; 95% CI: 1.18-7.86) and no effect in haplogroup T carriers (OR = 0.88; 95% CI: 0.39-2.00). We conducted sensitivity analyses using models adjusted for age only (Additional file 1: Table S8) or for age and education (Additional file 1: Table S9), but not for other covariates. Results from these models were consistent with those from the fully adjusted models.

To complete our analysis, we evaluated whether BC exposure was associated with differences in MMSE. Only a subset of the cohort participants had more than one cognitive assessment (n = 387) on which differences on MMSE could be calculated. In this analysis, clusters of mtDNA haplogroups did not show any main or modifier effects on the change of cognitive function (Additional file 1: Table S10-S11). A borderline effect of BC on change of cognition was found among haplogroup I carriers and haplogroup W carriers (Additional file 1: Table S12).

## Discussion

In the present investigation, we found that clusters of mitochondrial haplogroups modified the association between long-term exposure to traffic air pollution – defined as the 1-year average of BC levels before the baseline assessment – and cognition in a cohort of older men. We showed that the BC effect on low performance in MMSE scores was limited to Cluster 4 carriers, and possibly Cluster 1 carriers. No association between BC and low cognition was found among carriers of Cluster 2 and 3. To our knowledge, this is the first study to investigate and identify mitochondrial haplogroups and their clusters as modifiers of effects of environmental exposures on cognitive aging.

Due to unique population histories, mtDNA haplogroups vary substantially in different areas of the world and reflect the history of the female lineage through human migrations [9]. According to the mtDNA evolutionary tree, Cluster 4 is composed by the oldest mtDNA haplogroups I, X and W [17]. Only few studies have evaluated the functional differences between mitochondrial haplogroups. Haplogroups J has been associated with increased L-strand transcription and mtDNA copy number [18]. Macrohaplogroup N, ND3 and ATP6 may affect complex I activity, membrane potential, and Ca^11^ regulation [19].

Air pollution exposure has been recently linked with increased levels of mtDNA damage, as reflected in increased mtDNA copy number [20] and reactive species of oxygen (ROS) generation [21,22]. ROS have been implicated in inflammatory cytokine production, suggesting a connection between oxidative stress and inflammatory processes [23]. MtDNA haplogroups may also affect the coupling of the respiratory chain [24], which can result in increased endogenous ROS in mitochondria [25]. Recent work has demonstrated that mtDNA haplogroups modify the relationship of traffic-related air pollution exposures with systemic biomarkers of inflammation [26], suggesting a role of mitochondrial haplogroups on systemic inflammation. The impact of these mechanisms on cognitive function or in local inflammation in the central nervous system has not yet been characterized. However Cluster 4 has been associated with frontotemporal lobar degeneration in a previous study in Finland [11]. Within both Cluster 4 and Cluster 1, the non-synonymous/synonymous rate in mtDNA encoded genes has been shown to be higher than in the remaining European mtDNA haplogroup clusters [27]. The relative excess of nonsynonymous mutations in Clusters 4 and 1 has been suggested to play key roles in the risk of developing frontotemporal lobar degeneration [11]. In view of these findings, our results suggest that mtDNA clusters may be involved in determining the degree of damage in the central nervous system caused by environmental pollutants. MtDNA haplogroups might modulate exposure-induced inflammation and oxidative stress responses, which are both critical to determine loss of cognitive function throughout aging.

We recognize several limitations to our investigation. Our findings are based on a cohort of older men and may be generalized only to populations with similar age and gender characteristics. Additional studies are warranted to confirm our results among women and other ethnic group. Due to low proportions of most mtDNA haplogroups, we conducted our primary analysis on phylogenetic clusters. Larger studies are warranted to evaluate the effects of individual haplogroups within each cluster. The data used in the present study differ from our previous work in the NAS [3] in that we restricted the sample to study participants with mtDNA haplogroups and ethnicity information. Due to this restriction, we observed a less robust main effect of BC on cognitive function, possibly due, at least in part, to lower statistical power. We used geospatial models to estimate ambient BC and represent personal exposures of traffic-related pollution. Most error involved in evaluating air pollution exposures are of Berkson’s type. Simulation studies have shown this exposure misclassification is highly unlikely to bias away from the null and may rather lead to underestimation of air pollution effects [28]. Since BC concentration levels are spatially heterogeneous due to the numerous local (mobile) sources, measurement error in our BC exposure metric would likely reduce the true association. Thus, it is unlikely that this error would affect our conclusions.

## Conclusions

Our results indicate that mtDNA haplogroups may modify the effects of traffic particles on cognitive impairments in this population of elderly men. If confirmed, these results may provide a novel approach to determine susceptibility to the toxic effects of traffic pollution, as measured by BC, and contribute to growing understanding of the roles of mitochondria in the effects of environmental stressors.

## Abbreviations

ATP: Adenosine-5′-triphosphate; BC: Black carbon; BMI: Body mass index; CI: Confidence interval; IRB: Institutional review board; LUR: Land use regression model; MET-hr: Metabolic equivalent hours; MMSE: Mini-mental state examination; mtDNA: Mitochondrial DNA; NAS: Normative Aging Study; OR: Odds ratio; RNA: Ribonucleic acid; SD: Standard deviation; VA: US Department of Veterans Affairs.

## Competing interests

The authors declare that they have no competing interests.

## Authors’ contributions

EC performed the statistical analysis and drafted the manuscript. MCP contributed to perform the statistical analysis. DGC and MSG participated in data generation. MGW participated in developing the study concept and provided assistance in the interpretation of cognitive data. LH and SEA participated in developing the study concept and provided advice in performing the statistical analysis. PV initiated the study cohort and has conducted its follow-up. AS implemented cognitive testing in the cohort. JS developed exposure assessment strategies, directed the use of exposure data, and supervised statistical analysis. AB developed the study concept, contributed to the interpretation of the data and helped to draft the manuscript. All authors read and approved the final manuscript.

## Authors’ information

EC: Postdoctoral Research Fellow in Biostatistics. Department of Environmental Health, Harvard School of Public Health, Boston, MA, USA. MCP: PhD. Department of Environmental Health and Department of Epidemiology, Harvard School of Public Health, Boston, MA, USA. DGC: BS MS PhD. Research Associate. Cancer Research Center of Lyon, Lyon, France. MGW: Associate Professor of Environmental and Occupational Epidemiology. Department of environmental Health and Department of Epidemiology, Harvard School of Public Health, Boston, MA, USA. LH: Associate Professor in Preventive Medicine. Department of Preventive Medicine Northwestern University Feinberg School of Medicine, Chicago, IL, USA. SEA: PhD Department of Biostatistics, Harvard University, Boston, MA, USA. MSG: Postdoctoral Research Fellow. Department of Environmental Health, Harvard School of Public Health, Boston, MA, USA. PV: MD. VA Boston Healthcare System University Schools of Public Health and Medicine, Boston, MA, USA. AS: Research Professor. VA Boston Healthcare System University Schools of Public Health and Medicine, Boston, MA, USA. JS: Professor of Environmental Epidemiology. Department of environmental Health and Department of Epidemiology, Harvard School of Public Health, Boston, MA, USA. AAB: Mark and Catherine Winkler Associate Professor of Environmental Epigenetics Department of Environmental Health and Department of Epidemiology, Harvard School of Public Health, Boston, MA, USA.

## Supplementary Material

Additional file 1: Table S1 Eligible and not eligible participants. **Table S2.** Differences in exposure and outcome between eligible participants and participants without haplogroup information. **Table S3.** Distribution of MMSE performance by mitochondrial clusters and haplogroups. **Table S4.** Chi-Square Test of Association. **Table S5.** Effect of clusters on the low MMSE. **Table S6.** Effect for doubling in Black Carbon (BC) concentration on the natural scale on the low MMSE score, overall and by mitochondrial clusters. **Table S7.** Effect for doubling in BC concentration on the natural scale on the low MMSE score by individual haplogroups. **Table S8.** Effect for doubling in Black Carbon (BC) concentration on the natural scale on low MMSE score, overall and by mitochondrial clusters, adjusted for age and age and education. **Table S9.** Effect for doubling in Black Carbon (BC) concentration on the natural scale on low MMSE score, overall and by mitochondrial clusters, adjusted for age and age and education. **Table S10.** Effect of clusters on the change of MMSE. **Table S11.** Effect for doubling in Black Carbon (BC) concentration on the natural scale on the change of MMSE score, overall and by mitochondrial clusters. **Table S12.** Effect for doubling in BC concentration on the natural scale on the change of MMSE score by individual haplogroups. Click here for file
