# Supplementary material for: Mitochondrial haplogroups modify the effect of black carbon on age-related cognitive impairment
Source: Environ Health. 2014 May 30;13:42. doi: 10.1186/1476-069X-13-42 (PMC4049407; doi:10.1186/1476-069X-13-42)
Supplement: Additional file 1: Table S1 — Eligible and not eligible participants. Table S2. Differences in exposure and outcome between eligible participants and participants without haplogroup information. Table S3. Distribution of MMSE performance by mitochondrial clusters and haplogroups. Table S4. Chi-Square Test of Association. Table S5. Effect of clusters on the low MMSE. Table S6. Effect for doubling in Black Carbon (BC) concentration on the natural scale on the low MMSE score, overall and by mitochondrial clusters. Table S7. Effect for doubling in BC concentration on the natural scale on the low MMSE score by individual haplogroups. Table S8. Effect for doubling in Black Carbon (BC) concentration on the natural scale on low MMSE score, overall and by mitochondrial clusters, adjusted for age and age and education. Table S9. Effect for doubling in Black Carbon (BC) concentration on the natural scale on low MMSE score, overall and by mitochondrial clusters, adjusted for age and age and education. Table S10. Effect of clusters on the change of MMSE. Table S11. Effect for doubling in Black Carbon (BC) concentration on the natural scale on the change of MMSE score, overall and by mitochondrial clusters. Table S12. Effect for doubling in BC concentration on the natural scale on the change of MMSE score by individual haplogroups. [file 1476-069X-13-42-S1.pdf]

ADDITIONAL MATERIAL

Table S1. Eligible and not eligible participants

Units Selected from Sampling Frame	N
**Respondent, total**	1121
**Respondent not eligible**	
Experienced a stroke	17
**Unable to determine eligibility**	
Missing mtDNA haplogroup information	452
Missing covariates	47
Missing MMSE	0
Missing BC estimates	23
**Respondent screened and eligible, total**	582

Table S2. Differences in exposure and outcome between eligible participants and participants without haplogroup information.

Variables	Eligible participants (582)	Participants without haplogroup information (452)
BC_baseline - mean(SD)[NA]	0.59 (0.28) [0]	0.49 (0.31) [419]
MMSE - mean(SD) [NA]	26.89 (1.73) [0]	26.77 (2) [32]
MMSE ≤ 25 N(%) [NA]	232 (18.59%) [0]	165 (19.71%) [32]

Table S3. Distribution of MMSE performance by mitochondrial clusters and haplogroups.

Haplotypes	MMSE≤25	MMSE>26
**Cluster 1 (J or T)**		
Haplogroup J	26 (11.2%)	88 (8.7%)
Haplogroup T	26 (11.2%)	92 (9.1%)
**Cluster 2 (H or V)**		
Haplogroup H	23 (10.0%)	80 (7.9%)
Haplogroup V	98 (42.2%)	429 (42.2%)
**Cluster 3 (K or U)**		
Haplogroup K	24 (10.3%)	97 (9.5%)
Haplogroup U	14 (6.0%)	117 (11.5%)
**Cluster 4 (I, W or X)**		
Haplogroup I	10 (4.3%)	57 (5.6%)
Haplogroup W	2 (0.9%)	16 (1.6%)
Haplogroup X	9 (3.9%)	40 (3.9%)

Table S4. Chi-Square Test of Association.

Variables	MMSE (binary)	mtDNA Haplogroups
	p-value	p-value
**Age**	<.0001	0.6336
**Education**	<.0001	<.0001
**First Language**	0.0001	0.003
**Computer Experience**	<.0001	0.0002
**Physical Activity (MET-hr/week)**	<.0001	0.0648
**Alcohol (drinks/day)**	0.0204	0.0055
**Diabetes**	0.0278	0.0389
**Consumed Dark Fish (times/week)**	0.4965	0.0002
**Smoking Status**	0.0784	<.0001
**BMI (Kg/m^2)**	0.2749	0.0221
**Hypertension**	0.1804	0.0322
**Ethnicity**	0.0049	<.0001
**MtDNA Haplogroups**	0.2645	.

Table S5. Effect of clusters on the low MMSE.

Effect	OR[a,b]	95% CI[a,b]
**Cluster 1**	1.22	(0.78-1.91)
**Cluster 2**	Ref.	.
**Cluster 3**	0.84	(0.52-1.36)
**Cluster 4**	1.00	(0.52-1.91)

[a] Adjusted for age, education, matrilineal ethnicity, first language, computer experience, smoking, body mass index (BMI), physical activity, alcohol intake, diabetes, hypertension, dark fish consumption, percentage of the participant's census tract that is nonwhite, percentage of residential census tract adults with a college degree, indicator for first cognitive assessment, indicator for whether the participant was a part-time resident of the greater Boston area.
[b] Cluster 2 was taken as reference group.

Table S6. Effect for doubling in Black Carbon (BC) concentration on the natural scale on the low MMSE score, overall and by mitochondrial clusters.

BC effect in:	OR[a]	95% CI[a]	OR for the interaction term[a,b]	95% CI for the interaction term[a,b]
**All participants**	1.22	(0.95-1.56)		
**Cluster 1**	1.62	(0.90-2.91)	1.48	(0.77-2.86)
**Cluster 2**	1.10	(0.79-1.53)	Ref.	.
**Cluster 3**	0.97	(0.60-1.58)	0.89	(0.50-1.57)
**Cluster 4**	2.70	(1.30-5.59)	2.46	(1.10-5.48)

[a] Odds ratios (ORs) and 95% Confidence Intervals (CIs) adjusted for age, education, matrilineal ethnicity, first language, computer experience, smoking, body mass index (BMI), physical activity, alcohol intake, diabetes, hypertension, dark fish consumption, percentage of the participant's census tract that is nonwhite, percentage of residential census tract adults with a college degree, indicator for first cognitive assessment, indicator for whether the participant was a part-time resident of the greater Boston area.
[b] ORs and 95% CIs for the interaction term between clusters and BC concentration. The ORs express the modification of the relative odds for low MMSE associated with a doubling in BC concentrations for each of the clusters relative to the most common cluster, i.e. Cluster 2, taken as reference.

Table S7. Effect for doubling in BC concentration on the natural scale on the low MMSE score by individual haplogroups.

BC effect in:	OR[a]	95% CI[a]	OR for the interaction term[a,b]	95% CI for the interaction term[a,b]
**Cluster 1**				
**Haplogroup J**	3.04	(1.18;7.86)	2.89	(1.04;8.01)
**Haplogroup T**	0.88	(0.39;2.00)	0.84	(0.35;2.01)
**Cluster 2**				
**Haplogroup H**	1.40	(0.65;3.04)	1.33	(0.58;3.05)
**Haplogroup V**	1.05	(0.74;1.50)	Ref.	.
**Cluster 3**				
**Haplogroup K**	0.91	(0.53;1.57)	0.87	(0.46;1.63)
**Haplogroup U**	1.43	(0.56;3.60)	1.35	(0.51;3.61)
**Cluster 4**				
**Haplogroup I**	3.68	(0.92;14.73)	3.49	(0.83;14.78)
**Haplogroup X**	2.55	(1.05;6.20)	2.42	(0.93;6.28)
**Haplogroup W**	4.15	(0.53;32.73)	3.94	(0.49;31.41)

[a] Odds ratios (ORs) and 95% Confidence Intervals (CIs) adjusted for age, education, matrilineal ethnicity, first language, computer experience, smoking, body mass index (BMI), physical activity, alcohol intake, diabetes, hypertension, dark fish consumption, percentage of the participant's census tract that is nonwhite, percentage of residential census tract adults with a college degree, indicator for first cognitive assessment, indicator for whether the participant was a part-time resident of the greater Boston area.
[b] ORs and 95% CIs for the interaction term between mtDNA haplogroups and BC concentration. The ORs express the modification of the relative odds for low MMSE associated with a doubling in BC concentrations for each of the mtDNA haplogroup relative to the most common haplogroup, i.e. haplogroup V, taken as reference.

Table S8. Effect for doubling in Black Carbon (BC) concentration on the natural scale on low MMSE score, overall and by mitochondrial clusters, adjusted for age and age and education.

	**Age**				**Age and Education**			
**BC effect in:**	**OR**[a]	**95% CI**[a]	**OR for the interaction term**[a,b]	**95% CI for the interaction term**[a,b]	**OR**[c]	**95% CI**[c]	**OR for the interaction term**[c,b]	**95% CI for the interaction term**[c,b]
**All participants**	1.26	(1.00;1.58)			1.18	(0.94;1.48)		
**Cluster 1**	1.77	(0.99;3.18)	1.62	(0.84;3.14)	1.57	(0.87;2.83)	1.50	(0.78;2.91)
**Cluster 2**	1.09	(0.81;1.48)	Ref.	.	1.04	(0.78;1.40)	Ref.	.
**Cluster 3**	1.10	(0.69;1.74)	1.00	(0.57;1.75)	1.00	(0.62;1.62)	0.96	(0.55;1.68)
**Cluster 4**	2.16	(1.00;4.66)	1.97	(0.86;4.51)	2.18	(1.01;4.71)	2.09	(0.92;4.77)

[a] Adjusted for age
[b] Cluster 2 was taken as reference group.
[c] Adjusted for age and education.

Table S9. Effect for doubling in Black Carbon (BC) concentration on the natural scale on low MMSE score, overall and by mitochondrial clusters, adjusted for age and age and education.

	Age adjusted				Age and education adjusted			
**BC effect in:**	**OR[a]**	**95% CI[a]**	**OR for the interaction term[a,b]**	**95% CI for the interaction term[a,b]**	**OR[c]**	**95% CI[c]**	**OR for the interaction term[c,b]**	**95% CI for the interaction term[c,b]**
**Cluster 1**								
**Haplogroup J**	2.43	(1.14;5.19)	2.23	(0.97;5.11)	2.47	(1.02;5.99)	2.41	(0.93;6.21)
**Haplogroup T**	1.29	(0.5;3.31)	1.18	(0.44;3.2)	1.00	(0.42;2.38)	0.98	(0.39;2.46)
**Cluster 2**								
**Haplogroup H**	1.12	(0.51;2.45)	1.02	(0.44;2.4)	1.18	(0.59;2.35)	1.15	(0.53;2.47)
**Haplogroup V**	1.09	(0.78;1.52)	Ref.	.	1.03	(0.74;1.42)	Ref.	.
**Cluster 3**								
**Haplogroup K**	0.91	(0.55;1.52)	0.83	(0.45;1.53)	0.87	(0.53;1.44)	0.85	(0.47;1.55)
**Haplogroup U**	1.95	(0.81;4.69)	1.78	(0.7;4.57)	1.67	(0.65;4.29)	1.63	(0.61;4.4)
**Cluster 4**								
**Haplogroup I**	2.84	(0.73;10.96)	2.60	(0.65;10.44)	3.02	(0.77;11.86)	2.94	(0.72;12.02)
**Haplogroup X**	1.93	(0.68;5.54)	1.77	(0.59;5.34)	2.00	(0.73;5.46)	1.95	(0.68;5.61)
**Haplogroup W**	2.51	(0.92;6.85)	2.30	(0.80;6.60)	2.43	(0.87;6.82)	2.37	(0.81;6.96)

[a] Adjusted for age

[b] Haplogroup V was taken as reference.

[c] Adjusted for age and education.

Table S10. Effect of clusters on the change of MMSE.

Effect	Estimate[a,b]	95% CI[a,b]
**Cluster 1**	-0.11	(-0.58;0.36)
**Cluster 2**	Ref.	.
**Cluster 3**	-0.01	(-0.44;0.43)
**Cluster 4**	-0.09	(-0.58;0.40)

[a] Adjusted for age at the baseline, difference of age between the cognitive assessment and the baseline assessment, education, matrilineal ethnicity, first language, computer experience, smoking, body mass index (BMI), physical activity, alcohol intake, diabetes, hypertension, dark fish consumption, percentage of the participant's census tract that is nonwhite, percentage of residential census tract adults with a college degree, indicator for first cognitive assessment, indicator for whether the participant was a part-time resident of the greater Boston area.

[b] Cluster 2 was taken as reference group.

Table S11. Effect for doubling in Black Carbon (BC) concentration on the natural scale on the change of MMSE score, overall and by mitochondrial clusters.

BC effect in:	Estimate[a]	95% CI[a]	Estimate for the interaction term[a,b]	95% CI for the interaction term[a,b]
**All participants**	0.13	(-0.11;0.37)		
**Cluster 1**	-0.09	(-0.58;0.40)	-0.21	(-0.78;0.35)
**Cluster 2**	0.12	(-0.16;0.41)	Ref.	.
**Cluster 3**	0.31	(-0.49;1.11)	0.18	(-0.66;1.03)
**Cluster 4**	0.16	(-0.27;0.58)	0.03	(-0.47;0.53)

[a] Adjusted for age at the baseline, difference of age between the cognitive assessment and the baseline assessment, education, matrilineal ethnicity, first language, computer experience, smoking, body mass index (BMI), physical activity, alcohol intake, diabetes, hypertension, dark fish consumption, percentage of the participant's census tract that is nonwhite, percentage of residential census tract adults with a college degree, indicator for first cognitive assessment, indicator for whether the participant was a part-time resident of the greater Boston area.
[b] Cluster 2 was taken as reference group.

Table S12. Effect for doubling in BC concentration on the natural scale on the change of MMSE score by individual haplogroups.

BC effect in:	Estimate[a]	95% CI[a]	Estimate for the interaction term[a,b]	95% CI for the interaction term[a,b]
**Cluster 1**				
**Haplogroup J**	-0.09	(-0.54;0.37)	-0.15	(-0.70;0.39)
**Haplogroup T**	-0.24	(-1.38;0.90)	-0.31	(-1.50;0.88)
**Cluster 2**				
**Haplogroup H**	0.83	(-0.22;1.87)	0.76	(-0.31;1.83)
**Haplogroup V**	0.07	(-0.23;0.36)	Ref.	.
**Cluster 3**				
**Haplogroup K**	0.91	(-0.37;2.20)	0.85	(-0.47;2.17)
**Haplogroup U**	-0.30	(-0.90;0.30)	-0.36	(-1.01;0.28)
**Cluster 4**				
**Haplogroup I**	1.02	(-0.01;2.05)	0.96	(-0.12;2.03)
**Haplogroup X**	-0.16	(-1.06;0.75)	-0.22	(-1.17;0.72)
**Haplogroup W**	0.40	(-0.01;0.81)	0.34	(-0.15;0.83)

[a] Adjusted for age at the baseline, difference of age between the cognitive assessment and the baseline assessment, education, matrilineal ethnicity, first language, computer experience, smoking, body mass index (BMI), physical activity, alcohol intake, diabetes, hypertension, dark fish consumption, percentage of the participant's census tract that is nonwhite, percentage of residential census tract adults with a college degree, indicator for first cognitive assessment, indicator for whether the participant was a part-time resident of the greater Boston area.

[b] Haplogroup V was taken as reference.
